# “Who has ever loved a drug addict? It’s a lie. They think a ‘teja’ is as bad person”: multiple stigmas faced by women who inject drugs in coastal Kenya

**DOI:** 10.1186/s12954-018-0235-9

**Published:** 2018-05-25

**Authors:** Gitau Mburu, Sylvia Ayon, Alexander C. Tsai, James Ndimbii, Bangyuan Wang, Steffanie Strathdee, Janet Seeley

**Affiliations:** 1 Division of Health Research, Lancaster University, Lancaster, UK; 2 Kenya AIDS NGO Consortium, Nairobi, Kenya; 3 Chester M. Pierce, MD Division of Global Psychiatry, The Massachusetts General Hospital, Boston, USA; 4 International HIV/AIDS Alliance, Brighton, UK; 5 Division of Global Public Health, University of California San Diego School of Medicine, San Diego, USA; 6 Department of Public Health and Policy, London School of Hygiene & Tropical Medicine, London, UK

**Keywords:** Stigma, HIV, Harm reduction, Injecting drug use, Women who inject drugs, Kenya

## Abstract

**Background:**

A tenth of all people who inject drugs in Kenya are women, yet their social contexts and experiences remain poorly understood. This paper reports how multiple forms of stigma are experienced by women who inject drugs in coastal Kenya and the impact that they have on their ability to access essential health services.

**Methods:**

In 2015, in-depth interviews and focus group discussions were held with 45 women who inject drugs in two coastal towns. These data were supplemented with in-depth interviews with five individual stakeholders involved in service provision to this population. Data were analyzed thematically using NVivo.

**Results:**

Women who inject drugs experience multiple stigmas, often simultaneously. These included the external stigma and self-stigma of injection drug use, external gender-related stigma of being a female injecting drug user, and the external stigma of being HIV positive (i.e., among those living with HIV). Stigma led to rejection, social exclusion, low self-esteem, and delay or denial of services at health facilities.

**Conclusion:**

HIV and harm reduction programs should incorporate interventions that address different forms of stigma among women who inject drugs in coastal Kenya. Addressing stigma will require a combination of individual, social, and structural interventions, such as collective empowerment of injecting drug users, training of healthcare providers on issues and needs of women who inject drugs, peer accompaniment to health facilities, addressing wider social determinants of stigma and discrimination, and expansion of harm reduction interventions to change perceptions of communities towards women who inject drugs.

## Background

Injecting drug use is a significant driver of the global HIV epidemic [1]. In Kenya, 18.7% of people who inject drugs are HIV positive [2], which is three times the national prevalence of 5.6% [3]. In this context, gender vulnerabilities influence HIV acquisition. HIV prevalence is higher among women than men, both in the general population and among people who inject drugs [4, 5]. In addition, a marked geographic variation in HIV prevalence exists. Among injecting drug users, HIV prevalence is higher at the coast [2, 6].

To reduce social and health harms related to injecting drug use, a comprehensive package of harm reduction interventions is recommended for all persons who inject drugs. This package includes needle and syringe exchange programs (NSPs), opioid substitution therapy with methadone (OST), and prevention and treatment of a range of infectious diseases including HIV, viral hepatitis, tuberculosis, and sexually transmitted infections [7].

Despite the known benefits of harm reduction services for persons who inject drugs [8], provision of these interventions remains sub-optimal globally [9, 10]. In Kenya, the Ministry of Health endorsed the harm reduction approach in 2013 [11] and, in 2014, introduced NSP and OST in selected government facilities [12]. However, less than a fifth of an estimated 18,327 people who inject drugs in Kenya are being reached by the national NSP [3], access to OST with methadone is limited [13], and needle/syringe sharing is common [6].

In this context, drug use is generally stigmatized, and several authors have suggested that access to services could be restricted by the social perception of injecting drug use. Stigma is defined as the co-occurrence of labeling, stereotyping, separation, and discrimination in response to a discrediting attribute that is associated with loss of status [14, 15]. Through social and structural mechanisms, stigma reinforces existing inequalities and power imbalances such as those due to gender, ethnicity, physical characteristics, and so forth [16]. Enactments of stigma manifest as overt discrimination [14, 17], which may restrict stigmatized individuals’ access to important health services [18, 19].

Women who inject drugs often face gender and power inequalities [20]. In Kenya, women comprise of a tenth of all injecting drug users [21], yet they have received very limited attention to date [22]. There is limited understanding of these women's social contexts in Kenya, partly because injecting drug use has been relatively rare in sub-Saharan Africa compared to other global regions [23, 24]. Given the rising prevalence of injecting drug use in Kenya [23], we sought to unpack their lived experiences, explore how stigma could affect their access to health services, and recommend corrective actions.

We conducted a qualitative study whose aim, broadly, was to explore the needs and social contexts of women who inject drugs in coastal Kenya. We report ways in which women who inject drugs experience stigma and discrimination and the impacts that stigma and discrimination may have on their abilities to access health services. We propose potential programmatic and policy approaches that can mitigate the impact of stigma on service access.

## Methods

### Study design

In 2015, a combination of in-depth interviews and focus group discussions (FGDs) was conducted among women who inject drugs. These were complemented with in-depth interviews conducted with selected key informants involved in providing harm reduction services to this population.

### Setting

Data were collected from women who were accessing community-based harm reduction services in Mombasa and Kilifi. These services were being provided to injecting drug users through a partnership of three community-based organizations (CBOs): Kenya AIDS NGOs Consortium (KANCO), Reach out Centre Trust (REACH OUT), and the Muslim Education and Welfare Association (MEWA). Through outreach and linked drop-in services operated by these CBOs, injecting drug users were provided with clean needles, syringes, addiction counseling, HIV counseling and testing, condoms, pregnancy tests, and oral contraceptives. Clients who required additional services were referred to surrounding private and government facilities.

### Participant selection and recruitment

Participants included 45 women who inject drugs and five key stakeholders. Of the 45 women, 24 participated in in-depth interviews (12 at each site) and 21 participated in three focus group sessions (three sessions in Mombasa and one session in Kilifi). To be included, participants had to be older than 18 years of age, so that they could provide independent consent; be within the reproductive age bracket of 18–49 years; and have a history of injecting drugs within the 90 days preceding the study. Women were invited to participate in the study by outreach workers during the course of their routine outreach, and those who accepted the invitations based on availability were screened and scheduled for appointments. In consultation with representatives from the two CBOs, five key stakeholders were purposively sampled based on their roles, expertise, and political or community representation. The selected stakeholders included one community health worker, two outreach workers, one ministry of health official, and one outreach manager.

### Data collection

In-depth interviews and FGDs were conducted in private rooms at the CBOs or at key stakeholders’ offices by two experienced interviewers (SA and JN). In-depth interviews and FGDs explored drug use, sexual and reproductive health, HIV testing experiences, and access to outreach or conventional government health services. All in-depth interviews and FGDs were conducted in Swahili or English, based on participants’ preferences, were audio recorded, and lasted between 45 and 60 min. At the end of in-depth interviews and FGDs, basic socio-demographic data were collected from participants.

### Data analysis

A summary of socio-demographic data was generated using Microsoft Excel (Microsoft Corporation, Redmond, Washington). Interviews and FGDs were transcribed into English, and thematic analysis conducted [25]. Transcripts were imported into NVivo® [26], and codes were generated independently by GM and JN. Recurrent themes were identified while examining the data for nuances, similarities, and differences through constant comparison approach [27]. Final codes were categorized to generate themes related to women’s experiences of stigma and its impact on their access to health services.

### Ethical considerations

This research was conducted in accordance with the World Medical Association’s [28] provisions governing research with human subjects. Data were collected in private rooms, written informed consent was obtained, and participants retained the right to withdraw participation at any time. Unique codes were used to track participants in lieu of names or other identifying information, and the data were password-protected. Ethical approval was provided by the National Commission for Science Technology and Innovation (ref: P/15/8861/4510).

## Results

### Participant characteristics

The mean age of the 45 women was 28.5 years. Majority were single, with at least one child, without secondary education, and either unemployed or working in informal sector. On average, participants had used polydrugs for a period of 8.5 years, and had specifically injected drugs for 2.6 years. Overall, 29% were sex workers, and over two thirds (69%) were not using contraception. The main drug injected was heroin (Table 1).

**Table 1:** Participant characteristics

Characteristic	IDIs	FGDs	Total	Percent
Age (mean, years)	26.4	30.5	28.4	–
Number of children (mean)			1.6	–
Education				
None	4	4	8	18
Primary	13	10	23	51
Secondary	6	6	12	27
Post-secondary	0	1	1	2
Unknown	1	0	1	2
Marital status				
Married	5	3	8	18
Live in partner	7	5	12	27
Single	11	13	24	53
Unknown	1	0	1	2
Income source				
Casual labor	2	5	7	16
Food Kiosk/plaiting	3	2	5	11
Sex work	9	4	13	29
Peddling	1	2	3	7
Peer educator	0	1	1	2
Family or partner	3	1	4	9
Begging, hustling	5	6	11	24
Unknown	1	0	1	2
Contraception				
Yes	11	3	14	31
No	13	18	31	69
Drug use				
Duration using drugs (years)	7.8	9.1	8.5	–
Duration injecting (years)	3.3	2.0	2.6	–
Main drugs used				
Heroin	11	1	12	27
Heroin and other drugs^*^	11	15	26	58
Cocaine	1	3	4	9
Cocaine and other drugs^*^	1	2	3	6

^*^Varied combination of substances including Rhohypnol, khat, cigarettes, and alcohol

### Typologies and multiplicity of stigma

Our study found several forms of stigma. These were the stigmas of being a drug user, gender-related stigma of being a female injecting drug user, and the stigma of being HIV positive (i.e., among those living with HIV).

#### Self-stigma of women who inject drugs

Participants’ accounts indicated a self-directed shameful consciousness of being an injecting drug user. Internalization of stigma was often associated with low self-esteem. Indeed, some participants constructed these self-stigmatized identities in such a way as to make it impossible to have meaningful relationships, believing that they were not worth being loved:

You can meet someone who pretends to love and [desire] you. Who has ever loved a drug addict? It’s a lie. (Participant 10 Mombasa).

Self-stigmatization resulted from study participants internalizing the negative perceptions of injecting drug use within the study setting, where drug use was generally construed as resulting from a failure of individual morality. Injecting drug users were commonly referred as “teja” to which translates into “an injecting drug user” or “client” in a demeaning way. Not surprisingly, among the participants, the identity of being a drug user or “teja” was seen as shameful and regrettable. Hence, these stigmatized participants had an interest in concealing their stigmatized identities:

I usually don’t like people to know [that I inject drugs] because I feel shame. (Participant 12, Kilifi).

#### External stigmatization of injecting drug use in communities and health facilities

Although women experienced different degrees of stigmatization, they consistently perceived negative attitudes towards injecting drug use from their communities:

You know when you start using illicit drugs, obviously people who do not use will refuse to associate with you. No one will accept to walk with you when you are a drug addict. You will have to associate with your fellow drug users. (Participant 7, Mombasa).

Others stated that they were also stigmatized by their own family members:

From my own perspective, even the outreach workers are more concerned with us and love us even more than our families. The family contributes to our suffering as they say: “you are a drug addict; you are as good as dead”. If you try explaining anything to them, they see you as nothing but a drug addict. It pains. (Participant 10, Mombasa).

Participants described how this stigma was operationalized within a milieu of social dynamics and moralistic attitudes, conveying a notion of moral judgment:

They think a teja [drug user] is as bad person. (Participant 10, Mombasa).

This stigmatization was linked to moralistic judgements and suspicion, as drug users were generally viewed as likely to be petty criminals:

You can have a friend, maybe a neighbor, but if s/he knows, s/he will think: “so this one is an injecting drug user, aah it is over! Either thieves will steal from me or she will influence my children to snort or smoke [drugs]” Most of them in Mombasa are fond of that. (Participant 10, Mombasa).

Participants’ accounts suggested that although drug use was generally stigmatized, this stigma was accentuated among women. Accounts suggested that women perceived a unique stigma of drug use, because injecting drug use ran contrary to gendered norms of behavior. One stakeholder illustrated the prevalent negative perception of injection drug use by women, claiming that “it is a shame for a woman to be an injecting drug user” (Stakeholder 3, Kilifi).

Apart from experiencing it in communities, the stigma of injecting drug use was frequently experienced in healthcare settings. Asked about her experience with health care providers, one participant reported that “they despise us a lot” (Participant 10, Kilifi). Another commented that “they tell each other: ‘that is a drug user’” (Participant 5, Kilifi).

#### Stigma of being an HIV positive drug user

Our data provided insight into the cognitive and emotional experiences when the stigma of being a female drug user was layered over HIV-related stigma. Our data suggested that both can exist simultaneously. Participants who were HIV positive described perceiving HIV stigma, even from their fellow drug users, suggesting that their experiences as women who inject drugs and who lived with HIV were different from the experiences of women who inject drugs who were not HIV positive:

Now a fellow drug user tells you “Get out of here, didn’t we go with you to REACHOUT and got tested, and it was found that you are HIV positive?” (Participant 3, Mombasa).

These claims were confirmed by HIV negative participants, who constantly viewed HIV with stigmatizing suspicion. Their accounts suggested that being HIV positive was a different source of shame among women who injected drugs. In an illustrative comment, one participant suggested that she was very careful not to acquire HIV as “there is nothing [as] shameful as HIV” (Participant 12, Mombasa). As can be noted from this extract, her concerns were not necessarily related to the health effects of HIV infection but rather to the social perception of being HIV positive.

### Negative impact of stigma

Regardless of its source, stigmatization of women seemed to result in isolation and exclusion through prejudiced social processes and institutional practices. Responding to a question about the impact of stigma, one woman described how families and associates “abandon you and run away” (FGD 2, Mombasa) once she began injecting drugs. Referring to social distancing and its reasons, another opined that “people cannot agree to be with someone who is snorting or injecting drugs” (Participant 9, Kilifi). This distancing was also exemplified in the experiences of another participant, who asserted that her “best friend doesn’t use” and as a result their “friendship has reduced” (Participant 11, Kilifi).

Apart from social isolation, our data suggested that enactments of stigma—overt discrimination—served as a significant barrier to health service access. Participants characterized the actions of health providers as “scornful maltreatment” (Participant 10, Mombasa). Discrimination was common, whereby drug users were isolated and served last within health facilities:

Should they know that you are an addict, they send you backwards on the queue or tell you to go and come later. (Participant 10, Kilifi).

Participants’ narratives suggested that they were concerned about being identified or recognized as drug users within facilities, as this inevitably affected the quality of their interactions with health care providers, and ultimately delayed their services:

You get in the queue and you are the last one to be served, or they take you round from one place to the other once they know you are a drug user. (Participant 5, Kilifi).

Apart from existing negative perceptions of drug use, stakeholders suggested that “the health care workers did not understand why and how they needed to serve female drug users” (Stakeholder 3, Kilifi). Nevertheless, given women’s experiences, some of them opted not to seek health services. For one participant, being stigmatized at health facilities meant that she had “never gone back” (Participant 10, Kilifi). Yet, many women inevitably found themselves compelled by circumstances to seek health services, and in those situations, they strived to conceal their identities, so as to avoid being stigmatized or discriminated against:

I was forced to tell the truth that I was an addict. It was by luck that I met a good nurse. Had I have found the wrong one, I would have been insulted a lot, and not attended to. (Participant 10, Mombasa).

In many cases, participants opted to be accompanied by outreach workers in their visits to health facilities, so as to mitigate the effects of stigma. One participant asserted that “if you come along with an outreach worker, the health providers give you the required services but if you come alone, they may not attend to you” (Participant 10, Mombasa). Other participants suggested that the mediating role of CBOs extended to referral slips which were provided by outreach services:

Immediately they see this [referral] paper from REACHOUT they treat you with respect. If you don’t have one, you can be really mistreated, you can get there at 7 am and leave at even 5 pm, 8 pm or even 10 pm. (Participant 12, Mombasa).

## Discussion

In this qualitative study of 45 women who inject drugs and five stakeholders who were involved in the provision of harm reduction services, we report various experiences of stigma and discrimination among women who inject drugs in coastal Kenya. Specifically, this study found that several forms of external and self-stigma are concurrently experienced by these women. These include internal and external stigma of being a drug user, external gender-related stigma of being a female injecting drug user, and external stigma of being HIV positive among participants who were living with HIV.

The finding of self-stigma is consistent with other studies which have described internalized self-stigma among persons who inject drugs, which in turn leads to feelings of shame, low self-esteem, and reduced self-efficacy [29, 30]. In terms of external stigmatization within social and healthcare settings, other studies from India, Mexico, Vietnam, and the UK have also shown that women who inject drugs frequently experience severe stigma and social exclusion from their communities due to social disapproval of female injecting drug use [31–34]. Similar to these studies, our findings suggest that gendered norms of behavior operated in conjunction with stigma of drug use to exacerbate stigma faced by women who injected drugs.

On their part, HIV positive women who injected drugs experienced a simultaneous layering of multiple stigmas. In addition to experiencing stigma related to injecting drug use, HIV positive women perceived HIV-related stigma. HIV negative injecting drug users in our study also stigmatized their HIV positive colleagues, demonstrating that injecting drug users were themselves liable of stigmatizing others. In the UK, injecting drug users tended to stigmatize other injecting drug users they believed to be *worse* than them—primarily the homeless [35].

Our study shows that different forms of stigmatization of women who inject drugs are a potent barrier to their access and utilization of health services. Experiencing shame associated with one’s drug use can lead to avoidance of harm reduction [35] and general healthcare services [36]. In our study, poor access to services was mediated by poor interactions with, or overt discrimination by health providers. Other studies have shown that stigma is often a driver of problematic patient-provider relationships [37], which act in concert with isolation [38], and other healthcare barriers to limit drug users’ utilization of health services. Although this was not apparent in our study, layering of internalized stigma and HIV-related stigma also act simultaneously to prevent engagement of injecting drug users with HIV care in other settings [18]. These findings suggest that unless the stigma confronting women who inject drugs is mitigated, the health of this marginalized population is likely to get worse.

### Implications

Addressing different forms of stigma will be essential to mitigate their negative impacts on women’s ability to access health services. These can be addressed at individual, social, and structural levels. At the individual level, interventions that support reversal of internalized stigma are required. Unfortunately, few interventions for effectively combating internalized stigma exist [39]. However, research among stigmatized populations has shown that collectivization and peer-based support approaches can assist in coping and confronting self and external stigma, by harnessing collective self-efficacy, and providing an environment to transform self-esteem [40, 41]. As such, increasing recruitment of female peers and forming peer support groups of women who inject drugs would be critical to overcoming self-stigma in the study context. It can also strengthen women’s negotiating power to access social and health entitlements, as has been the case among other stigmatized populations [42].

At the social level, addressing moralistic judgements and attitudes that reinforce conservative, but often inequitable stigmatization of women who inject drugs will be essential. Working with community and religious leaders as an avenue to tackle stigma at the coast might be useful. Resistance from the local county government and community members might be encountered, given the prevalent social perception of drug use in coastal Kenya. However, community-based advocacy and outreach to religious and other community leaders has been shown to soften hard community stances and to reduce community discrimination against drug users in Vietnam [43]. The success of such an approach would be in combining education to communities about the possible harmful effects of adhering to prevalent stigmatizing attitudes towards women who inject drugs, while informing them of the benefits of harm reduction in increasing social functioning and health of injecting drug users. Thus, the local leadership needs to see drug use as partly a symptom of social problems that confront women, rather than an issue of simple individual choice that abstinence and prohibition can solve. Because community support and commitment is essential in combating social stigma against drug users [43, 44], community-based programs (for example through CBOs) that can reach communities with anti-stigma messages will need to be geographically expanded, politically supported, and financially resourced.

At the structural level, interventions to eliminate stigmatization in healthcare settings will be crucial. The need to train healthcare providers on issues and needs of injecting drug users in the Kenyan coast has already been noted, given the rising number of women who inject drugs, who will require health services [22]. Involvement of women in training and service delivery could be particularly useful as it has been shown to facilitate understanding of their needs and a change of negative attitudes among healthcare providers in Tanzania [45]. Providing health workers with a broader understanding of injecting risk behaviors has also been a critical part of responses to injecting drug use in the USA, Thailand, and other countries with established harm reduction programs [46, 47]. For optimal impact, the expanded training will need to be accompanied by an increased provision of the globally recommended comprehensive package of harm reduction services [7]. On their part, healthcare staff must resist the reproduction of stigma in health care settings and should learn and use drug user’s language in interactions with them. Creating opportunities for multiple encounters and making a personal connection have been shown to generate trust between drug users and health providers in Tanzania [45], and such an approach could allow health providers in our study's context to be familiar with, and change their attitudes towards injecting drug users. In addition, our study suggests that peer accompaniment and support to navigate health facilities should be continued as a strategy to mediate communication with health providers and reduce experiences of stigma among women who inject drugs. In future, online stigma reduction training of health providers using hypothetical scenarios and concerns around injecting drug users, which was found to have a positive impact on stigmatizing attitudes among Australian health providers [48], could be adapted for our study’s context.

However, addressing the social and health systems drivers of stigma need to be linked to wider social determinants of stigma and discrimination. Redirecting government drug policy from being exclusively abut clinical treatment to also include community and socially oriented harm reduction interventions will be crucial in mitigating stigma, as it provides an avenue to strengthen employment [49], livelihoods and skills development [22], progressive policing [50], legal support, and violence mitigation within a rights-based approach [20, 43, 51]. These interventions, combined with widespread expansion of services to all drug users, will likely trigger a change in social attitudes towards women who inject drugs.

### Limitations

Several limitations of our findings should be noted. Our study involved participants who were in contact with community-based harm reduction services, whose accounts and experiences of stigma may differ from other women. It is indeed possible that our study may be underestimating the impact of stigma among women who inject drugs in Kenya, because our samples were already accessing pyschosocial and other harm reduction services. Our study did not include clinical service providers, who might have provided useful triangulating information regarding their attitudes towards women who inject drugs. Nevertheless, this study contributes to the knowledge base regarding the social contexts of women who inject drugs in Kenya, upon which tailored interventions can be based, and future studies be built upon.

## Conclusions

Without purporting to fully understand or to oversimplify the contexts and experiences of stigma, it is clear that women who inject drugs in the Kenyan coast often self-stigmatize, face stigma of injecting drug use in social contexts and health care facilities, and are discriminated by healthcare providers, which deters their access and utilization of health services particularly harm reduction, HIV and reproductive health services. In addition, these stigmas are overlaid on HIV-related stigma among women who are living with HIV. To overcome the multiple forms of stigma simultaneously experienced by participants in this study and ensure that tailored gender-sensitive interventions are available to them, a range of specific individual-, social-, and structural-level interventions will need to be implemented.
